# Thyrotoxic Hypokalemic Periodic Paralysis: A Success Story of a Diagnostic Challenge

**DOI:** 10.7759/cureus.14553

**Published:** 2021-04-19

**Authors:** Mohsin Nazir, Malika Hameed, Rizwana Shehzad

**Affiliations:** 1 Anesthesiology, Aga Khan University Hospital, Karachi, PAK

**Keywords:** hyperthyroidism, hypokalemic periodic paralysis, hypokalemia, paralysis

## Abstract

Thyrotoxic hypokalemic periodic paralysis (THPP) is a rare but life-threatening complication of hyperthyroidism seen predominantly in males. It is generally characterized by hypokalemia and skeletal muscle paralysis requiring intensive care admission. Hypokalemia occurs due to the massive intracellular shift of potassium because of the hyperactivity of the sodium-potassium adenosine triphosphates pump (Na^+^ K^+^ ATPase). Its differential diagnosis from the other common causes of hypokalemic paralysis is essential to provide targeted therapy. We present a rare case of THPP in a female patient with no prior history of thyroid disease. THPP in this patient was precipitated by trauma and emotional stress, which served as a diagnostic challenge. Both hypokalemia and elevated levels of T3 and T4 are important diagnostic features during the acute episode. Treatment of THPP includes nonselective beta-blockade, which prevents the shift of intracellular potassium, and potassium replacement. THPP is curable once a euthyroid state is achieved.

## Introduction

Thyrotoxic hypokalemic periodic paralysis (THPP) is a lethal complication of hyperthyroidism leading to muscle weakness requiring ventilatory support, cardiac arrhythmias, and death. It is generally characterized by hypokalemia and skeletal muscle paralysis. Hypokalemia occurs due to the massive intracellular shift of potassium because of the hyperactivity of the sodium-potassium adenosine triphosphates pump (Na+ K+ ATPase). It is most commonly reported in the Asian populations. Among the Asians with hyperthyroidism, the incidence of THPP is around 2%, while among non-Asians with hyperthyroidism, the incidence of THPP is 0.1%-0.2% [1]. Predisposing factors for THPP are trauma, surgery, emotional stress, high carbohydrate and salt intake, rest after strenuous unaccustomed exercise, cold exposure, alcohol, and drugs like diuretics, estrogen, laxatives, steroids and amphotericin B, etc. [2].

We present a case of a 26-year-old female patient with no prior history of thyroid disease who was eventually diagnosed to be suffering from THPP. The diagnosis served as a challenge for all the teams involved in the care of this patient.

## Case presentation

A 26-year-old female patient with no known co-morbidities presented in the ER of a tertiary care hospital with complaints of lower limb weakness and respiratory difficulty since morning. She was the victim of domestic violence with no psychiatric issues. Two days prior to admission, she faced domestic violence with blunt trauma to her back. With that history, cervical or thoracic cord injury was suspected. There was no significant past medical and drug history.

On examination, she was responsive with a normal sensory system, decreased power in both lower limbs (1/5), plantars were down going with depressed deep tendon reflexes. She was having difficulty in breathing and was using accessory muscles of respiration since morning. Cranial nerves were intact and there were no signs of autonomic instability. Respiratory examination revealed grade IV dyspnea and decreased, but symmetrical chest expansion. On auscultation, decreased breath sounds were present bilaterally. Her cardiovascular examination was unremarkable and ECG was normal.

Chest X-ray and MRI of the cervical and thoracic spine were done, which were unremarkable. There was no sign of cervical and thoracic cord injury and/or compression at any level. Echocardiography was done, which was normal. Complete blood count, serum electrolytes, magnesium, urea, creatinine, random blood sugar, and coagulation profile were within normal limits except serum potassium and bicarbonate levels. There was severe hypokalemia with a serum potassium level of 1.6 mmol/L and low bicarbonate levels of 12.7 mmol/L. Arterial blood gases showed a pH of 7.23, PO2 of 130.5 mmHg, and PCO2 of 30.4 mmHg. Blood and urine culture/sensitivity were sent to rule out underlying infection and an empiric antibiotic (Inj. Amikacin 500 mg Q12H) was started with a suspicion of urinary tract infection. Serum creatine phosphokinase (CPK) was raised with values of 520 U/L (normal value: up to 195 U/L) while creatine kinase myocardial band (CKMB) was slightly increased with the value of 10.2 IU/L (normal value: 0-10 IU/L).

The patient was shifted to the intensive care unit (ICU) for advanced care. Invasive arterial blood pressure, central venous pressure, respiratory rate, arterial oxygen saturation, urine output, and temperature were monitored. Initially, non-invasive ventilation (NIV) was applied, which was not tolerated by the patient, so it was converted to invasive mechanical ventilation. Intravenous potassium chloride infusion was initiated immediately at a rate of 30-40 mEq/hr through the central venous line with hourly urine output monitoring and four hourly serum potassium level was checked. Hypokalemic periodic paralysis was suspected. On the third day of admission, serum potassium levels improved to 3.8 mmol/L, but there was no improvement in muscle power and ventilatory efforts.

The internal medicine team was on board from the first day of admission. When no improvement was noted after two days, the case was thoroughly discussed with the team of Internal Medicine consultants who suggested further investigations, including an electromyogram, an MRI brain with multiple sclerosis (MS) protocol, and a thyroid function test. Electromyogram and MRI were done which were unremarkable. Thyroid function test showed increased levels of free T4, 2.31 ng/dL (lab normal range: 0.71-1.85 ng/dL), low T3, 0.66 ng/ml (lab normal range: 0.79-1.49 ng/ml) and low thyroid-stimulating hormone (TSH), 0.01 mIU/L (lab normal range: 0.35-4.94 mIU/L). Based on lab results, a rare condition of THPP which has a male predominance was suspected in the patient. Anti-thyroid medication (propylthiouracil 50 mg Q8H) was started and continued. During her stay in ICU, ventilatory parameters remained within acceptable limits. No significant abnormalities in gas exchange and airway pressures were observed. On the fourth day of admission, muscle power started to recover dramatically and within the next two days, the patient became ready for tracheal extubation. She was extubated on the sixth day of ICU admission with complete muscle power recovery and normal laboratory parameters.

## Discussion

The muscular weakness in THPP ranges from minor weakness to complete flaccid paralysis. Bladder, bowel, and sensory functions are generally not affected [2,3]. Usually, the proximal muscles are more severely affected than the distal muscles. The acute attack initially involves the lower limbs, followed by girdle muscles and subsequently upper limbs [4]. Respiratory muscles are rarely involved, but total paralysis of respiratory, bulbar, and ocular muscles has been reported in a severe attack [5]. The pathogenesis of THPP is poorly understood. Hypokalemia is the typical finding with raised thyroid hormones. Increased adrenergic responses and high circulating levels of insulin and thyroid hormones enhance Na K ATPase activity [5,6]. This leads to hypokalemia and subsequent periodic paralysis. Some patients may develop metabolic acidosis. The treatment of THPP includes immediate supplementation of potassium. Potassium chloride (KCL) can be given intravenously and orally or both. The dose of KCL varies among patients and can be titrated according to renal clearance and cardiovascular status [4]. Overly aggressive treatment with potassium can result in rebound hyperkalemia and requires caution. Non-selective beta-blockers, like propranolol, can be considered to block catecholamines' effect on ion channels [7]. Anti-thyroid drugs and radioactive iodine should also be initiated in patients with Graves’ disease, multinodular goiter, and thyroid toxic adenoma. Precipitating factors including stress, undue exertion, and high carbohydrate and salt diet should be avoided [4,8].

Epidemiologically, the case of THPP in the female gender is an extremely rare occurrence. The male-to-female ratio ranges from 17:1 to 70:1 despite the fact that hyperthyroidism is more common in females [7]. In our case, the patient was an Asian female with no previous history of hypokalemia, hyperthyroidism, and/or skeletal muscle weakness. Although she had no prior history of thyroid disease, she had emotional stress and vague history of trauma to the back, both of which are the predisposing factors for developing THPP. The history of trauma to the cervical region led us to suspect cervical cord injury. Later on, she was managed successfully according to the defined approach to treat THPP.

## Conclusions

THPP is a rare but life-threatening condition if untreated. Early recognition, admission to the appropriate healthcare facility (i.e., high-dependency unit [HDU] and/or ICU), and specific treatment initiation are the keys to success. Supportive patient management like ventilatory support, acid-base balance, and hemodynamic management are crucial interventions for successful treatment.
